# Retrospektive Auswertung elektronisch erhobener Patientenfragebögen einer universitären Schmerzambulanz mit dem painDETECT®-Fragebogen

**DOI:** 10.1007/s00482-022-00677-3

**Published:** 2022-11-24

**Authors:** N. Foadi, I. Winkelmann, M. Rhein, M. Karst

**Affiliations:** 1 Klinik für Anästhesiologie und Intensivmedizin, Schmerzambulanz, Medizinische Hochschule, Carl-Neuberg-Str. 1, 30625 Hannover, Deutschland; 2 Klinik für Psychiatrie, Sozialpsychiatrie und Psychotherapie, Medizinische Hochschule Hannover, Hannover, Deutschland

**Keywords:** Chronischer Schmerz, Ambulantes Kollektiv, Statistische Datenanalyse, Therapieerweiterung, Chronic pain, Outpatient collective, Statistical data analysis, Treatment extension

## Abstract

**Hintergrund und Ziel der Arbeit:**

Psychometrische Tests können wichtige Hinweise für Diagnostik und Verlauf bei chronischen Schmerzpatienten geben. Zwischen 2008 und 2018 wurde in der Schmerzambulanz der Medizinischen Hochschule Hannover (MHH) das elektronische System painDETECT® eingesetzt. Die vorliegende retrospektive Arbeit verfolgte das Ziel, die mittels painDETECT® erfassten Daten zur Schmerzsymptomatik und die in Anspruch genommenen Therapieverfahren der in einem Zeitraum von 15 Monaten untersuchten Patientenkohorte zu evaluieren.

**Material und Methoden:**

Es erfolgte eine statistische Analyse der erfassten schmerzbezogenen Parameter zum Zeitpunkt des Studieneinschlusses sowie im Therapieverlauf unter Einbeziehung der Ambulanzakten.

**Ergebnisse:**

Es konnten Baseline-Daten von 459 Patienten (66 % Frauen) ausgewertet werden. Die häufigsten Krankheitsbilder waren Schmerzen im Bereich der Wirbelsäule, Kopf- und Gesichtsschmerzen und somatoforme Störungen bei meist langjähriger Vortherapie. Ca. 40 % zeigten Hinweise für neuropathische Schmerzkomponenten oder zentrale Sensibilisierung. Bei einer durchschnittlichen Schmerzintensität von VAS 6 (0–10) lag ein überwiegend hoher Chronifizierungsgrad vor. Ca. ein Drittel zeigte eine hochgradige schmerzbedingte Funktionseinschränkung. Etwas mehr als die Hälfte zeigte Hinweise auf eine klinisch relevante Depression. Ca. 80 % wiesen klinisch relevante Schlafstörungen auf. Daten zur Verlaufsbeobachtung lagen für 145 Patienten (31,6 %) vor. Der Anteil der Patienten, die eine nichtmedikamentöse Therapieform erhielten, erhöhte sich im Beobachtungszeitraum um 44,1 % (Physiotherapie) bzw. um 24,1 % (Psychotherapieverfahren). Die Verwendung von Koanalgetika stieg im Verlauf um ca. 30 %.

**Diskussion:**

Im ambulanten Setting kann bei hochgradig chronifizierten Schmerzpatienten eine Therapieerweiterung gelingen. Enge strukturelle Vernetzungen mit den Kliniken für Rehabilitationsmedizin und für Psychosomatik und Psychotherapie der MHH können hierfür eine günstige Voraussetzung sein.

## Abstract (en)

**Background and objective:**

Psychometric tests can provide important information for diagnostics and progression in chronic pain patients. Between 2008 and 2018, the electronic system painDETECT® was used in the outpatient pain clinic of the Hannover Medical School (MHH). The aim of this retrospective study was to evaluate the pain symptomatology data recorded using painDETECT® and the treatment procedures used in the patient cohort examined over a period of 15 months.

**Material and methods:**

A statistical analysis of baseline and follow-up data was performed. The analysis comprised pain-related parameters recorded by use of the painDETECT® system as well as outpatient records.

**Results:**

Baseline data of 459 patients (66% women) could be evaluated. The most common clinical pictures were spinal pain, headache, facial pain, and somatoform disorders, mostly with many years of previous treatment. Approximately 40% showed evidence of neuropathic pain components or central sensitization. With a mean pain intensity of VAS 6 (0–10), a predominantly high degree of chronicity was present. Approximately one third showed a high degree of pain-related functional impairment. Slightly more than half showed evidence of clinically relevant depression. Approximately 80% showed clinically relevant sleep disturbances.

Follow-up data were available for 145 patients (31.6%). The proportion of patients receiving a nonpharmacological form of treatment increased by 44.1% (physical therapy) and by 24.1% (psychotherapeutic procedures) during the observation period. The use of co-analgesics increased by approximately 30% over the course.

**Conclusion:**

In the outpatient setting, an extension of treatment can be successful for high-grade chronic pain patients. Close structural networking with the clinics for rehabilitation medicine and for psychosomatics and psychotherapy at the MHH can be a favorable prerequisite for this.

## Hintergrund und Fragestellung

Mit dem painDETECT®-Projekt wurde 2004 eine zentrale deutschlandweite Erhebung epidemiologischer Daten chronischer Schmerzpatienten initiiert. Der vorliegenden Arbeit liegen Daten einer Patientenkohorte der universitären Schmerzambulanz der MHH (Medizinischen Hochschule Hannover) zugrunde, die mittels des painDETECT®-Fragebogensystems erhoben wurden. Diese retrospektive Untersuchung verfolgte das Ziel, die Schmerzsymptomatik dieser Patientenkohorte zu charakterisieren und Einflüsse medikamentöser und nichtmedikamentöser Therapieformen auf schmerzassoziierte Parameter zu erfassen.

## Einleitung

Chronische Schmerzen sind mit einer Vielzahl somatischer und psychosozialer Faktoren verbunden, die dieses multidimensionale Krankheitsbild kennzeichnen. Wie im biopsychosozialen Modell von Engel beschrieben, ist entsprechend eine diagnostisch und therapeutisch breite Sichtweise notwendig [1]. Mithilfe standardisierter Fragebögen können schmerzbezogene Beschwerden systematisch erfasst und dokumentiert werden. Hierzu zählen neben quantitativen Angaben wie der Schmerzintensität und der Schmerzschwelle qualitative Merkmale wie die Funktionsbeeinträchtigungen durch Schmerzen im Alltag.

Ab August 2004 wurden 500 Personal-Digital-Assistants-Handheld-Taschencomputer, die eigens mit dem elektronischen painDETECT®-Fragebogen ausgestattet worden waren, an 465 schmerztherapeutische Einrichtungen in Deutschland verteilt [2]. Neben Schmerzparametern sind im painDETECT®-Fragebogen unter anderem Angaben zu Depressivität, Angst, Schlafstörungen und der alltäglichen Funktionalität integriert [2]. Bei den Patienten der MHH-Schmerzambulanz kam der elektronische painDETECT®-Fragebogen von 2008 bis 2018 zum Einsatz. Die zu Beginn dieses Zeitraums (22.10.2008 bis 28.01.2010) akquirierten Patientendaten wurden in der vorliegenden Untersuchung analysiert.

## Studiendesign und Untersuchungsmethoden

**Figure 1:**
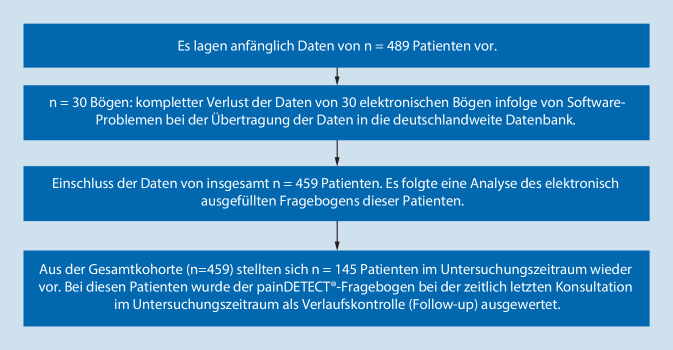

Die Datenerfassung und -auswertung erfolgte mittels des elektronischen Systems painDETECT®. Einmal im Quartal wurden mittels des elektronischen painDETECT®-Fragebogens psychometrische Befunde jedes Patienten der Schmerzambulanz der MHH erhoben. Seit Oktober 2008 werden die erfassten Patientendaten alle 12 bis 15 Monate in eine deutschlandweite Pooldatenbank (Institut StatConsult) übertragen und durch das Institut StatConsult in SPSS überführt. Für diese Analyse und Übertragung der zunächst pseudonymisierten und später anonymisierten Daten lag die schriftliche Einwilligung der Patienten vor [3]. Nach Einholen des positiven Ethikvotums der lokalen Ethikkommission wurden in dieser Studie SPSS-Daten von insgesamt 459 Patienten der Schmerzambulanz der MHH retrospektiv erfasst (Abb. 1). Neben dem Zeitpunkt des Einschlusses in die Studie (Zeitpunkt 1) wurde die im Untersuchungszeitraum zeitlich letzte Vorstellung (Follow-up) ausgewertet.

Zur Erfassung der Schmerzintensität wurde die visuelle Analogskala (VAS; 0–10 Punkte) verwendet.

Es erfolgte eine detaillierte deskriptive Analyse der Ergebnisse der in Tab. 1 aufgeführten Fragebögen ([2]; Tab. 1).

**Table 1:** 

Variable	Fragebögen
	Bezeichnung
Vorliegen neuropathischer Schmerzen	PainDETECT Questionnaire (PD‑Q; [4])
Chronifizierungsstadium	Mainzer Pain Staging System (MPSS; [5])
Depressive Symptomatik, Angst, Panikstörung	Patient Health Questionnaire Depression (PHQ‑D; [6])
Schmerzbedingte Funktionseinschränkungen	Pain Disability Index (PDI; [7])
Psychovegetative Beschwerden	Beschwerdenliste nach von Zerssen (B‑L; [8])
Schlafverhalten	Medical Outcome Studies Sleep Scale (MOS Sleep Scale; [9])
Funktionseinschränkungen bei alltäglichen Tätigkeiten	Funktionsfragebogen Hannover – Rückenschmerzen^a^ (FfbH‑R; [10])
Gradeinteilung von Rückenschmerzen	Schmerzgrading nach Kohlmann et Raspe^a^ [11]
Chronifizierungsrisiko bei Rückenschmerzen	Heidelberger Kurzfragebogen – Rückenschmerzen^a^ (HKF-R10; [12])
Krankheitsaktivität von Spondylarthropathien	Bath Ankylosing Spondylitis Disease Activity Index^a^ (BASDAI; [13])
Kopfschmerzstatus	Essener Kopfschmerzfragebogen^a^ [14]
Funktionelle Beeinträchtigung durch Kopfschmerzen	Migraine Disability Assessment^a^ (MIDAS; [15])

^a^Vor Aushändigung des Taschencomputers wurden die Patienten gefragt, ob sie unter Rücken- und/oder Kopfschmerzen leiden. Nur bei Patienten, die dies bejahten, wurden die in der Tabelle markierten Bögen (^a^) in die elektronische Befragung mit aufgenommen

Die Patienten wurden darüber hinaus gefragt, ob sie folgende therapeutische Maßnahmen erhalten hatten:

- Medikamente zur Schmerzreduktion und Verbesserung des Nachtschlafs. Die Angaben wurden hierauf weiter unterteilt in Nichtopioidanalgetika, Opioidanalgetika, Koanalgetika und Sedativa.
- Physiotherapie (aktiv und/oder passiv)
- Psychotherapie (hierunter wurden Verhaltenstherapie, Schmerzbewältigung im Gruppengespräch, psychodynamische Therapie sowie das Lernen von Entspannungstechniken subsumiert)
- Transkutane elektrische Nervenstimulation (TENS)
- Akupunktur

## Statistische Analyse

Die statistische Analyse wurde mit dem Statistikprogramm IBM-SPSS (Firma IBM, München, Deutschland), Version 24.0 für Windows, durchgeführt. Wurden Rangzahlen zwischen 2 Gruppen miteinander verglichen, kam der Mann-Whitney-Test zum Einsatz (bei mehr als 2 Gruppen: Kruskal-Wallis-Test). Wurden Veränderungen einer Variablen in einer Gruppe zwischen 2 Zeitpunkten betrachtet, wurde der Wilcoxon-Test verwendet. *p* ≤ 0,05 galt als Signifikanzschwelle, ab der von einem relevanten Ergebnis ausgegangen wurde.

Für die Berechnung der Einflüsse von physiotherapeutischen und psychotherapeutischen Interventionen auf die subjektive Schmerzwahrnehmung durch die Patienten wurden relevante Faktoren in einem gemischten linearen Modell zusammengeführt. Als Faktoren wurden die Zeitpunkte der Befragung (Baseline und Follow-up) sowie die physiotherapeutischen und psychotherapeutischen Interventionen im Untersuchungszeitraum gewählt (Physio- bzw. Psychotherapie im Zeitraum der Baseline-Befragung; Physio- bzw. Psychotherapie im Zeitraum der Follow-up-Befragung – jeweils Physio- bzw. Psychotherapie erhalten [ja/nein]). Die dabei errechneten geschätzten Randmittel („estimated marginal means“ [EMM]) für den PDI-Wert der einzelnen Untergruppen wurden für die Mehrfachbestimmung des Schmerzwerts nach Benjamini et al. [16] korrigiert.

## Ergebnisse

### Rückmeldungen zur Handhabung des Handgeräts

Auf die Frage nach der Handhabung des Handheld-Taschencomputers antwortete die Mehrzahl (79,8 %) der Patienten, dass das Gerät einfach zu bedienen sei, unabhängig davon, ob sie den Fragebogen zum ersten oder zweiten Mal durchgeführt haben. 13,5 % der Studienteilnehmer gaben einen mittleren Schwierigkeitsgrad an, 4,0 % bemängelten eine schwierige Handhabung. 2,7 % konnten den Handcomputer nur mit permanenter fremder Hilfe bedienen. Mit steigendem Alter der Patienten wurde die Bedienbarkeit signifikant schlechter bewertet. Jüngere Patienten im Alter bis 49 Jahre fanden in 88,7 % die Handhabbarkeit einfach, unter den Befragten mit einem Alter von mindestens 50 Jahren stuften nur noch 72,8 % die Handhabung als einfach ein.

## Charakterisierung des Patientenkollektivs und der Schmerzsymptomatik

### Geschlecht und Alter der Patienten

**Figure 2:**
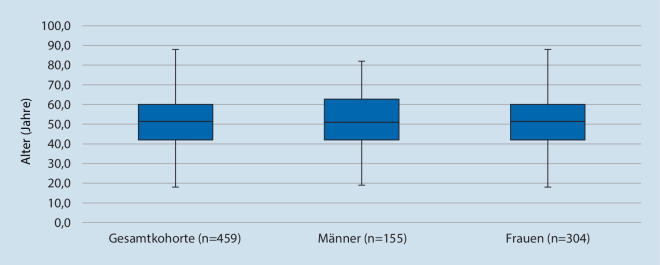

**Figure 3:**
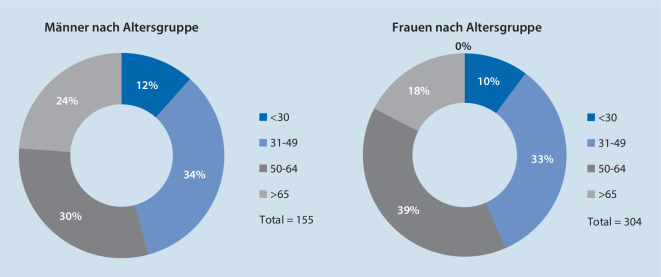

Der Frauenanteil überwog in der untersuchten Kohorte mit 66,2 %. Das durchschnittliche Alter im Gesamtkollektiv lag bei 51,1 ± 14,9 Jahren (Abb. 2). Die 50- bis 65-Jährigen hatten mit 36,2 % den größten Anteil, während die mittlere Altersgruppe der 31- bis 49-Jährigen mit 33,6 % vertreten war. In der Altersgruppe der bis 30-Jährigen befanden sich mit 10,6 % die wenigsten Patienten. 19,6 % der Studienteilnehmer waren 65 Jahre und älter. Abb. 3 zeigt die Verteilung der Altersgruppen bei beiden Geschlechtern.

### Therapiedauer

Insgesamt haben 350 Patienten Angaben darüber gemacht, wie lange sie bereits aufgrund von Schmerzen in ärztlicher Behandlung waren. Über ein Drittel der eingeschlossenen Patienten (*n* = 127, 36,2 %) war bei Studieneinschluss 61 Monate oder länger aufgrund von Schmerzen in ärztlicher Behandlung. Bei knapp einem Viertel der Patienten (*n* = 85, 24,3 %) lag die vorangegangene Behandlungsdauer unter einem Jahr. Von 449 Patienten, die Angaben über bisherige Therapeutenkontakte machten, hatten 398 Patienten (88,6 %) vor Studieneinschluss einen bis 8 Therapeutenkontakte (2,3 ± 1,9; Median 2).

### Schmerzhäufigkeit

Insgesamt machten 449 Patienten eine Angabe darüber, wie häufig sie unter Schmerzen litten. Unter diesen lag der Anteil mit täglich vorhandenen Schmerzen bei 88,7 % (*n* = 398). Wöchentlich auftretende Schmerzen wurden von 22 (4,9 %) sowie gelegentliche Schmerzen von 23 Patienten (5,1 %) beschrieben.

**Figure 4:**
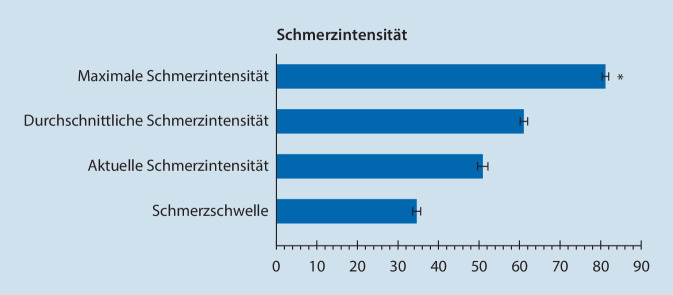

Tab. 2 und 3 sowie Abb. 4 und 5 zeigen in Übersicht die Auswertungsergebnisse der painDETECT®-Fragebögen zum Zeitpunkt des Studieneinschlusses.

**Table 2:** 

Merkmal/Schmerzfragebogen	Summenscore	Anzahl vorliegender Patientenangaben
	„mean“ ± SD	
*Depressive Störungen*		
PHQ-9-Fragebogen	11,1 ± 5,8	445
*Funktionsbeeinträchtigung durch die Schmerzen*		
PDI	35,2 ± 15,8	436
*Psychovegetative Beschwerden*		
Beschwerdescore nach von Zerssen	28,2 ± 13,3	426
*Funktionskapazität bei alltäglichen Tätigkeiten*		
FFbH – Rückenschmerzen	52,9 ± 24,7	107
< 60 %: klinisch relevante Funktionsbeeinträchtigung		
*BASDAI-Score*	48,1 ± 22,5	55
*MIDAS-Score*	61,9 ± 35,4	59

*PHQ-9* Patient Health Questionnaire Depression, *PDI* Pain Disability Index, *FFbH* Funktionsfragebogen Hannover, *BASDAI* Bath Ankylosing Spondylitis Disease Activity Index, *MIDAS* Migraine Disability Assessment

**Table 3:** 

Merkmal/Schmerzfragebogen	Anzahl betroffener Patienten	Gesamtanzahl vorliegender Patientenangaben
	(% der in der rechten Spalte angegebenen Gesamtanzahl)	(*n*)
*Vorliegen neuropathischer Schmerzen* gemäß PD-Q-Fragebogen		451
0–12 (< 15 % Wahrscheinlichkeit, dass neuropathische Schmerzen vorliegen)	169 (37,7 %)	
13–18 (keine eindeutige Aussage möglich)	98 (21,9 %)	
≥ 19 (neuropathische Schmerzen sehr wahrscheinlich [> 90 %])	181 (40,4 %)	
*Funktionsbeeinträchtigung durch die Schmerzen*		436
PDI	PDI-Score < 45: 302 (69,3 %)	
	PDI-Score ≥ 45: 134 (29,3 %)	
*Chronifizierungsstadium*		413
MPSS – Stadium I	53 (12,8 %)	
MPSS – Stadium II	185 (44,8 %)	
MPSS – Stadium III	175 (42,4 %)	
*Depressivität nach PHQ‑D*		445
Keine depressive Symptomatik (< 5 Punkte)	53 (11,9 %)	
Milde depressive Symptomatik (5–9 Punkte)	148 (33,3 %)	
Mittelgradige depressive Symptomatik (10–14 Punkte)	115 (25,8 %)	
Schwere depressive Symptomatik (≥ 15 Punkte)	129 (29,0 %)	
*Panikstörung* gemäß PHQ‑D		445
Keine Panikstörung	400 (89,9 %)	
Panikstörung	45 (10,1 %)	
*MOS-Schlafskala*		63
Optimaler Schlaf (7 oder 8 h Schlaf/Nacht)	13 (20,6 %)	
Kein optimaler Schlaf (< 7 h Schlaf/Nacht)	50 (79,4 %)	
*Chronifizierungsrisiko bei Rückenschmerzen* gemäß HKF-R10		62
≤ 2,5, höchstwahrscheinlich keine Chronifizierung	1 (1,6 %)	
> 2,5 und ≤ 8, Patient chronifiziert zu 70 % nicht	2 (3,2 %)	
> 8 und < 28, keine Aussage möglich	14 (22,6 %)	
≥ 28 und < 37, Patient chronifiziert zu 70 %	5 (8,1 %)	
≥ 37, Patient chronifiziert höchstwahrscheinlich	40 (64,5 %)	
*Schmerzgrading nach Kohlmann/Raspe*		103
0 (keine aktuellen Rückenschmerzen)	8 (7,8 %)	
1 (Rückenschmerzen ohne erhöhte Schmerzintensität und ohne ausgeprägte Funktionseinschränkung)	24 (23,3 %)	
2 (Rückenschmerzen mit erhöhter Schmerzintensität oder ausgeprägter Funktionseinschränkung)	70 (68,0 %)	
3 (Rückenschmerzen mit erhöhter Schmerzintensität und ausgeprägter Funktionseinschränkung)	1 (1 %)	
*BASDAI-Score*		55
BASDAI-Score < 4 (keine oder geringe Krankheitsaktivität)	18 (32,7 %)	
BASDAI-Score ≥ 4 (hohe Krankheitsaktivität)	37 (67,3 %)	
*Kopfschmerzstatus* gemäß Essener Kopfschmerzfragebogen		64
Migräne ohne Aura	53 (82,8 %)	
Diagnose Migräne mit Aura	20 (31,3 %)	
Episodische Migräne	32 (50,0 %)	
Chronische Migräne	16 (25,0 %)	
Spannungskopfschmerz	22 (34,4 %)	
Episodischer Spannungskopfschmerz	6 (9,4 %)	
Chronischer Spannungskopfschmerz	16 (25,0 %)	
Adäquater Medikamentenkonsum	42 (65,6 %)	
Diagnose Medikamentenmissbrauch	21 (32,8 %)	
Trigeminoautonomer Kopfschmerz	9 (14,1 %)	
Diagnose nicht klassifizierbarer Kopfschmerz	1 (1,6 %)	
*MIDAS*		61
Grad I 0–5 Punkte, wenig oder keine Beeinträchtigung	19 (31,1 %)	
Grad II 6–10 Punkte, geringe Beeinträchtigung	5 (8,2 %)	
Grad III 11–20 Punkte, mäßige Beeinträchtigung	6 (9,8 %)	
Grad IV > 21 Punkte, schwere Beeinträchtigung	31 (50,8 %)	

*PD-Q* PainDETECT Questionnaire, *PDI* Pain Disability Index, *MPSS* Mainzer Pain Staging System, *PHQ-D* Patient Health Questionnaire Depression, *MOS* Medical Outcome Studies Sleep Scale, *HKF* Heidelberger Kurzfragebogen, *BASDAI* Bath Ankylosing Spondylitis Disease Activity Index, *MIDAS* Migraine Disability Assessment

**Figure 5:**
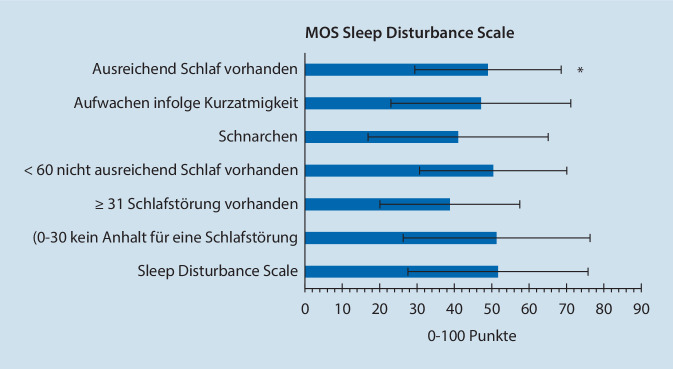

### Schmerzlokalisation

**Figure 6:**
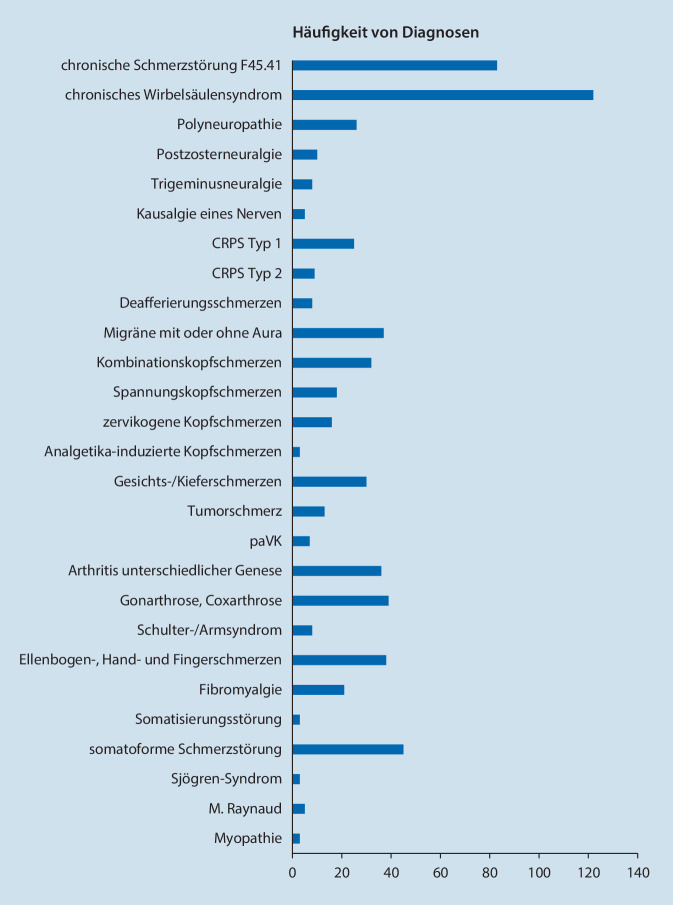

Bei der Schmerzzeichnung im elektronischen painDETECT®-Fragebogen konnten Patienten mehrere Lokalisationen aufführen, an denen sie Schmerzen haben. 139 Patienten (30,3 %) gaben an, unter Schmerzen in mehr als einer Körperregion zu leiden. Etwas mehr als die Hälfte der Patienten (*n* = 236; 51,4 %) beklagten Schmerzen in 2 verschiedenen Regionen, 73 Patienten (15,9 %) benannten Schmerzen in 3 und 11 Patienten (2,4 %) an 4 Regionen. 351 Patienten (76,5 %) zeichneten Kopfschmerzen ein. Rückenschmerzen gaben 246 Patienten (53,6 %) an. 161 Patienten (35,1 %) führten Gelenk- und Muskelschmerzen auf. Abb. 6 gibt einen Überblick über die Häufigkeiten schmerzbezogener Diagnosen.

## Veränderungen im Zeitverlauf

### Funktion und Schmerz im Zeitverlauf

Die folgenden Angaben beziehen sich ausschließlich auf das Patientenkollektiv, das mindestens 2 Konsultationen im Untersuchungszeitraum hatte: Zeitpunkt 1 = Zeitpunkt des Studieneinschlusses, Follow-up = letzte Konsultation im Untersuchungszeitraum. Von den 459 eingeschlossenen Patienten lagen im Follow-up Daten von 145 Patienten (31,6 %) vor.

### PHQ-D

Bei insgesamt 130 Patienten waren vollständige Angaben zum PHQ‑D in diesem Kollektiv vorhanden. Der Anteil von Patienten, bei denen gemäß dem PHQ-D-Fragebogen keine depressive Symptomatik detektiert wurde, betrug 9,2 % zum Zeitpunkt 1 und 15,4 % beim Follow-up (*p* < 0,001). Der Anteil von Patienten mit Verdacht auf milde depressive Symptomatik sank von 43 % (Zeitpunkt 1) auf 33,1 % beim Follow up (*p* < 0,001).

### PDI

In Bezug auf die Funktionsbeeinträchtigungen durch die Schmerzen im Alltag (PDI) war zum Zeitpunkt des Follow-ups im Vergleich zu Zeitpunkt 1 eine Verbesserung ersichtlich. 128 Patienten füllten in dieser Kohorte den PDI-Bogen komplett aus. Bei 33,6 % dieser Patienten lag bei Zeitpunkt 1 ein PDI-Summenwert von ≥ 45 vor. Bei der letzten Konsultation im Untersuchungszeitraum sank der Anteil der Patienten mit einem Summenwert ≥ 45 auf 25,8 % ab (*p* < 0,001).

Insbesondere die Patientengruppe, die sowohl im Zeitraum der Baseline-Befragung als auch beim Follow-up Physiotherapie erhielt, hatte gemäß der Berechnung mittels der geschätzten Randmittel nach Benjamini et al. [15] signifikant niedrigere PDI-Scorewerte (*p* < 0,001; Abb. 3).

### Physiotherapeutische Behandlungsverfahren

**Figure 7:**
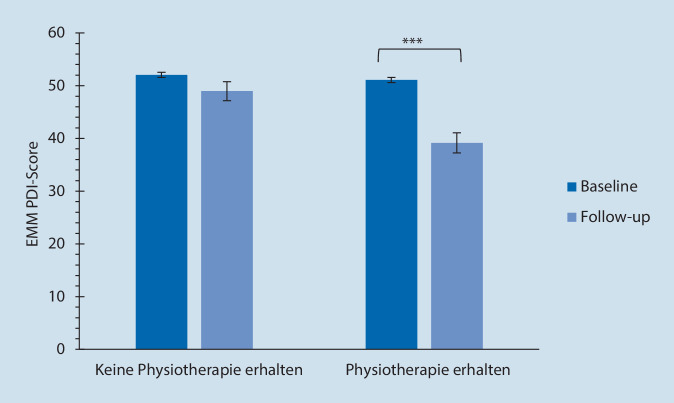

Im zeitlichen Verlauf (Vergleich Zeitpunkt 1 und Follow-up) stieg der Anteil an Patienten, die physiotherapeutische Maßnahmen erhielten, von 35,2 % auf 79,3 % an (*p* < 0,001). 67,6 % der Patienten erhielten hierbei eine aktive und 71 % eine passive Physiotherapie. Die Differenz der Werte für die Schmerzschwelle (VAS-Wert, unter dem der Schmerz als erträglich eingestuft wird) beim Follow-up im Vergleich zu Zeitpunkt 1 ergab, dass sich die Schmerzschwelle bei denjenigen, die Physiotherapie erhielten, im Verlauf anhob (*p* = 0,050). Die Patienten, die aktive Physiotherapie erhielten, zeigten beim Follow-up eine höhere Schmerzschwelle als zu Beginn (*p* = 0,050). Bei physiotherapeutischer Behandlung im Zeitraum von Baseline und Follow-up sanken die PDI-Messwerte im Vergleich zwischen diesen beiden Zeitpunkten, während bei der Gruppe ohne Physiotherapie kein Unterschied feststellbar war (Abb. 7, multiple T‑Tests mit einer Korrektur nach Benjamini et al. [15]; Physiotherapie bei Baseline: *p* < 0,001, df = 310; Physiotherapie bei Baseline und Follow-up: *p* < 0,001, df = 72; keine Physiotherapie: *p* = 0,293, df = 530; Tab. 4).

**Table 4:** 

Hauptdiagnose	*n* (%)
Rückenschmerzen	34 (46,0)
Kopfschmerzen	11 (14,9)
CRPS	11 (14,9)
Chronische Polyarthritis	5 (6,8)
Neuropathie	3 (4,1)
Fibromyalgie	3 (4,1)
Arthrose	3 (4,1)
Tumorschmerz	2 (2,7)
Somatoforme Schmerzstörung	2 (2,7)

*CRPS* Komplexes Regionales Schmerzsyndrom

### Psychotherapeutische Behandlungsverfahren

Im Follow-up erhöhte sich der Anteil von Patienten, die Psychotherapie erhielten, von 13,1 % auf 37,2 % und die Ablehnungsrate gegenüber psychotherapeutischen Verfahren reduzierte sich von 12,4 % auf 8,3 % (*p* < 0,001). Der Anteil von Patienten, denen Entspannungstechniken vermittelt wurden, verfünffachte sich im Follow-up-Zeitraum (von 2,1 % auf 11,7 %, *p* = 0,001). Der Anteil von Patienten, die eine Schmerzbewältigungsgruppentherapie absolvierten, stieg von 0,7 % (Zeitpunkt 1) auf 4,8 % (Follow-up; *p* < 0,001).

**Figure 8:**
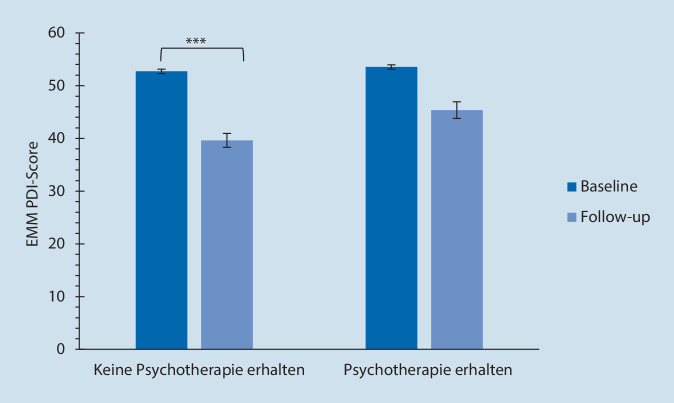

Bei der Analyse der Einflüsse von Psychotherapie wurde im Therapieverlauf eine nicht signifikante Verringerung des PDI-Scores gemäß der Berechnung nach Benjamini et al. [15] festgestellt. Hierbei zeigte sich in der Gruppe ohne Psychotherapie eine signifikante Abnahme des PDI-Scores beim Follow-up (Abb. 8).

**Figure 9:**
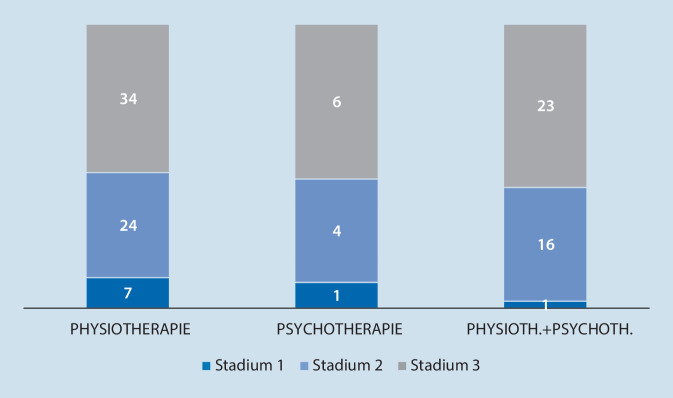

In Tab. 4 und 5 sind die Schmerzdiagnosen der Patienten aufgeführt, die im Follow-up jeweils physiotherapeutische beziehungsweise physiotherapeutische und psychotherapeutische Behandlungen erhielten. Abb. 9 stellt das Chronifizierungsstadium dieser Patientengruppen dar.

**Table 5:** 

Hauptdiagnose	*n* (%)
Rückenschmerzen	12 (28,6)
Kopfschmerzen	12 (28,6)
Fibromyalgie	8 (19,1)
Somatoforme Schmerzstörung	4 (9,5)
Chronische Polyarthritis	3 (7,1)
Neuropathie	1 (2,4)
CRPS	1 (2,4)
Kraniomandibuläre Dysfunktion	1 (2,4)

*CRPS* Komplexes Regionales Schmerzsyndrom

Bei Patienten, die sowohl Psycho- als auch Physiotherapie erhielten, zeigte sich eine, wenn auch nicht signifikante, Tendenz der Abnahme des PDI-Scores im Follow-up im Vergleich zu Patienten, die weder Psycho- noch Physiotherapie erhielten (*p* = 0,091; Tab. 5; Abb. 9).

### Pharmakotherapie

#### Verschreibung von Schmerzmitteln

Zum Zeitpunkt des Studieneinschlusses wurden etwa der Hälfte der Patienten (*n* = 230, 50,1 %) die Schmerzmittel vom Hausarzt verschrieben, danach folgten Schmerztherapeuten (*n* = 100, 21,8 %) und Orthopäden (*n* = 77, 16,8 %; Mehrfachantworten waren möglich). In Eigenregie aus der Apotheke bezogen 34 Patienten (7,4 %) ihre Medikation, wobei es sich hierbei um frei verkäufliche Schmerzmittel aus der Gruppe der Nichtopioidanalgetika handelte. Es wurden im zeitlichen Verlauf mehr Koanalgetika (z. B. antihyperalgetisch wirkende Antidepressiva) verordnet. Die Zahl von Patienten, die Koanalgetika einnahmen, lag zum Zeitpunkt 1 bei 67 und beim Follow-up bei 113 (es lagen von *n* = 138 Patienten vollständige Angaben vor). Die Einnahme von Sedativa (z. B. Benzodiazepine) zeigte einen Rückgang im zeitlichen Verlauf: Von 13 Patienten, die Sedativa einnahmen, reduzierte sich die Zahl im Follow-up auf 3 Patienten. Es ergab sich kein signifikanter Einfluss der Pharmakotherapie auf den PDI, die Schmerzintensitätswerte (akuter, durchschnittlicher, maximaler Schmerz) und die Schmerzschwelle (jeweils *p* > 0,300).

### Ergänzende Therapieverfahren

Zum Zeitpunkt des Studieneinschlusses hatten 17,2 % der Gesamtkohorte ergänzende Verfahren wie Akupunktur, therapeutische Lokalanästhesie oder TENS erhalten. Im Subkollektiv mit Follow-up-Konsultation nahm der Anteil an mit Akupunktur (6,2 % auf 11 %) und TENS (4,8 % auf 16,6 %) behandelten Patienten zu (Vergleich Follow-up mit Erstvorstellung; jeweils *p* < 0,001 für Akupunktur und TENS).

## Diskussion

Etwa zwei Drittel der Studienpopulation waren Frauen. Die häufigsten Krankheitsbilder waren Schmerzen im Bereich der Wirbelsäule, Kopf- und Gesichtsschmerzen und somatoforme Störungen. Ca. 40 % zeigten gemäß PDQ Hinweise für neuropathische Schmerzkomponenten oder zentrale Sensibilisierung. Knapp 90 % wiesen einen Chronifizierungsgrad von MPSS II oder III auf. Die durchschnittliche Schmerzintensität lag bei VAS 6 (0–10). Mindestens ein Drittel zeigte eine hochgradige schmerzbedingte Funktionseinschränkung. Etwas mehr als die Hälfte zeigte Hinweise auf eine klinisch relevante Depression. Ca. 80 % zeigten klinisch relevante Schlafstörungen. Ca. 90 % waren teilweise über viele Jahre hinweg vortherapiert.

Im Follow-up während der Behandlung in der Schmerzambulanz stieg im Vergleich zum Beginn der Analyse derjenige Anteil an Patienten, der Physiotherapie und/oder Psychotherapie in Anspruch nahm, signifikant an. Zum Zeitpunkt des Follow-ups nahmen annähernd dreimal so viele Patienten Psychotherapie in Anspruch im Vergleich zum Beginn der Studie. Der Anteil von Patienten, die eine Schmerzbewältigungsgruppentherapie durchliefen, stieg annähernd auf die siebenfache Anzahl. Signifikante Verbesserungen ergaben sich im Verlauf insbesondere bei der Funktionalität (PDI) und bei den Depressionsscores, sofern initial eine milde Ausprägung vorlag. Nachteilig sedativ wirkende Medikamente konnten im Behandlungsverlauf abgesetzt werden. Diejenigen Patientengruppen, die nur Psychotherapie oder Psychotherapie in Kombination mit Physiotherapie erhalten hatten, waren gemäß dem Chronifizierungsgrad MPSS stärker betroffen im Vergleich zu denjenigen Patienten, die nur Physiotherapie erhalten hatten. Dies könnte ein Grund dafür sein, dass die beschriebenen Verbesserungen in der Funktionalität und in der Ausprägung der Depression in der Physiotherapiegruppe deutlicher ausfielen.

Dieser Verlauf kann auf eine erfolgreiche Psychoedukation des untersuchten Patientenkollektivs hinweisen, in der es gelungen ist, die Bedeutung des biopsychosozialen Modells in der Schmerzmedizin zu vermitteln und die Motivation für entsprechende psychosoziale und aktive physiotherapeutische Ansätze zu erhöhen.

Dabei ermöglicht die enge Zusammenarbeit der MHH-Schmerzambulanz mit der Klinik für Rehabilitationsmedizin und der Klinik für Psychosomatik und Psychotherapie eine ambulante zeitnahe interdisziplinäre Behandlung chronischer Schmerzpatienten. Zusammen mit der Klinik für Rehabilitationsmedizin finden wöchentlich gemeinsame Sprechstunden statt. Innerhalb jeweils kurzer Zeit erhalten Patienten der Schmerzambulanz einen Termin in der psychosomatischen Poliklinik der MHH. Dort findet eine ausführliche Evaluation und Beratung statt, in der eine ambulante Weiterbetreuung oder eine stationäre oder teilstationäre Behandlung angeboten wird. Ein niedrigschwelliges Angebot stellen 14-tägige Gruppentherapiesitzungen zur Schmerzbewältigung dar. Durch diese koordinierten Strukturen gelingt eine enge und zeitnahe Zusammenarbeit mit diesen relevanten Fachgruppen. Eine kürzlich durchgeführte Befragung unter ambulant tätigen Schmerzmedizinern zeigte, dass die Mehrzahl der Befragten diesbezüglich unzufrieden ist [17]. Dabei kann der schnelle Beginn einer psychotherapeutischen Behandlung die Motivation der chronischen Schmerzpatienten für diesen Therapieansatz fördern [18].

Die stringente Psychoedukation ist so bedeutsam, da die Wahrnehmung von Schmerzen nicht nur durch den sensorischen Input, sondern auch durch kontextbasierte Vorhersagen geprägt wird [19]. Z. B. können körperbezogene Ängste dazu führen, dass harmlose somatosensorische Informationen als schmerzhaft interpretiert und erlebt werden [19, 20]. Insbesondere Gehirnareale, die affektiv-motivationale Inhalte repräsentieren, sind an diesem Schmerzerleben beteiligt [21]. Aus diesem Grund gelten Psychoedukation und kognitive Verhaltenstherapie als primäre Ansätze in einer solchen Situation [22]. Dabei können schon relativ kurze Interventionen zu therapeutisch relevanten Veränderungen führen [23, 24].

Eine Intensivierung der ambulanten Schmerztherapie stellt die stationäre interdisziplinäre multimodale Schmerztherapie (IMST) dar. Hierbei handelt es sich um eine „gleichzeitige, inhaltlich, zeitlich und in der Vorgehensweise aufeinander abgestimmte umfassende Behandlung von Patienten mit chronifizierten Schmerzsyndromen“, in die verschiedene Therapieverfahren in aufeinander abgestimmter Form eingebunden sind [25]. Das von allen beteiligten Fachgruppen gemeinsam getragene Therapieziel ist die physische und psychische (Re‑)Aktvierung auf dem Boden eines eigenverantwortlichen Umgangs mit den chronischen Schmerzen [25].

Die Versorgungsstruktur der ambulanten Schmerzmedizin ist in Deutschland allerdings bislang nicht für ein solches multimodales Therapieprogramm ausgelegt. Mit der Vereinbarung für eine ambulante multimodale Komplexbehandlung für Patienten mit chronischen Schmerzen haben 2019 die Kassenärztliche Bundesvereinigung (KBV), die Kassenärztlichen Vereinigungen und der Berufsverband der Ärzte und Psychologischen Psychotherapeuten in der Schmerz- und Palliativmedizin in Deutschland (BVSD) dazu ein Anfangssignal gesetzt [17]. Dabei sind die Kriterien für Struktur und Inhalt ambulanter multimodaler Behandlungsprozesse aber noch nicht definitiv geklärt [26].

Die Stärken dieser Arbeit liegen in der konsekutiven Erfassung einer großen Patientengruppe in einem natürlichen klinischen Setting über einen Zeitraum von 15 Monaten, in der Untersuchung einer Vielzahl von Parametern mittels des painDETECT®-Fragebogens sowie in der Beobachtung von Verlaufsdaten.

Die Schwächen dieser Arbeit stellen das retrospektive Design dar, Limitierungen des genutzten Fragebogensystems, die Heterogenität der Patientenklientel und die teilweise geringen Patientenzahlen im Follow-up. Vor diesem Hintergrund sind kausale Assoziationen nicht möglich.

Zusammenfassend zeigen die Daten überwiegend hoch chronifizierte und langjährig vorbehandelte Schmerzpatienten, bei denen es bei entsprechendem Angebot gelungen ist, auch physiotherapeutische und/oder psychosoziale Therapieansätze zu implementieren. Im Verlauf konnten Verbesserungen in Bezug auf die Funktionalität und Ausprägung von Depression erreicht werden.
